# Supplementary material for: Mutational Landscape of Autism Spectrum Disorder Brain Tissue
Source: Genes (Basel). 2022 Jan 24;13(2):207. doi: 10.3390/genes13020207 (PMC8871846; doi:10.3390/genes13020207)
Supplement: Supplementary file 1 [file genes-13-00207-s001.zip › genes-1556693-table s1.pdf]

Supplementary Table S1: Examination of expression profiles of predicted damaging mutations

	Germline		
	Pre-natal	Early childhood	Adult
ASD (Y/N)	117/49	106/60	106/60
Ctrl (Y/N)	68/24	63/29	63/29
OR	0.84	0.81	0.81
95% CI	0.48 – 1.49	0.47 – 1.40	0.47 – 1.40
	Post-zygotic		
	Pre-natal	Early childhood	Adult
ASD (Y/N)	8/3	7/4	7/4
Ctrl (Y/N)	4/5	4/5	4/5
OR	3.33	2.19	2.19
95% CI	0.51 – 21.58	0.36 – 13.23	0.36 – 13.23
